# The mediating role of the home environment in relation to parental educational level and preschool children’s screen time: a cross-sectional study

**DOI:** 10.1186/s12889-017-4694-9

**Published:** 2017-09-02

**Authors:** Suvi Määttä, Riikka Kaukonen, Henna Vepsäläinen, Elviira Lehto, Anna Ylönen, Carola Ray, Maijaliisa Erkkola, Eva Roos

**Affiliations:** 1 Folkhälsan Research Center, Samfundet Folkhälsan, Topeliuksenkatu 20, 00250 Helsinki, Finland; 2 Department of Food and Environmental Sciences, University of Helsinki, P.O. Box 66, -00014 Helsinki, FI Finland; 3 Clinicum, Department of Public Health, University of Helsinki, P.O. Box 66, -00014 Helsinki, FI Finland

**Keywords:** Sedentary lifestyle, Socioeconomic factors, Home environment, Screen time, Preschool children

## Abstract

**Background:**

Previous studies suggest that preschoolers from low socioeconomic backgrounds engage in more screen time. Still, the factors in the social and physical home environment driving these differences in preschool children’s screen time are poorly understood. This study examines potential home environment mediators in the associations between parental educational level and preschoolers’ screen time.

**Methods:**

A total of 864 children aged 3–6 years and their parents participated in a cross-sectional DAGIS study in 2015–2016. Parents recorded their children’s screen time in a diary (*N* = 823). For the analyses, the daily average screen time at home was calculated. Parental questionnaires (*N* = 808) assessed educational level and eight social and physical environment factors in the home (i.e., descriptive norm for children’s screen time, parental screen use in front of children, parental importance for limiting children’s screen time, parental attitude toward societal pressures for children’s screen time, access to screens at home, parental self-efficacy for limiting children’s screen time, satisfaction of children’s screen time, and rules for limiting children’s screen time). Parental education was grouped into low, middle, and high education. The associations were tested by conducting mediation analyses adjusted by season and children’s sex and age. The significant mediators in the single-mediator models were included in the final multiple-mediator models.

**Results:**

Of the potential eight mediators, the following four had a significant indirect association: descriptive norm for children’s screen time, parental screen use in front of children, parental importance for limiting children’s screen time, and parental attitude toward societal pressures for children’s screen time. Parents with high education had lower descriptive norm and used fewer screens in front of children compared to parents with middle or low education, and in turn, these factors were associated with less screen time among children from parents with a higher education level. Parents with high education placed greater importance on limiting children’s screen time and felt less societal pressures about children’s screen time compared to parents with low education, and in turn, these factors were associated with less screen time among children from parents with a higher education level.

**Conclusions:**

Our study recognized multiple modifiable mediators in the associations between parental education and preschool children’s screen time. When aiming to diminish socioeconomic status differences in preschool children’s screen time, the focus should be on parental role models, attitudes, and norm related to children’s screen time.

## Background

Excessive screen time has several influences on preschool children’s (aged 3–5 years) health and wellbeing; therefore, several national guidelines include recommendations on limiting preschool children’s daily screen time to one hour [1, 2]. Still, according to several recent studies [3–5], the average daily screen time for preschoolers exceeds the recommended levels. Concurrent evidence exists that children from low socioeconomic status (SES) backgrounds engage in more screen time than children from higher SES backgrounds [6], and the preschool children from low SES backgrounds tend to have higher risks of exceeding the screen-time recommendations [7–9]. Excessive amounts of screen time are a public health concern because of detrimental health associations. Excessive screen time, particularly television viewing, is associated with increased risks of obesity and weight gain, poorer cognitive and social skills, reduced fitness, and lower psychosocial wellbeing among children aged 3–5 years [10–12]. Recognizing factors that explain the association between SES and screen time is therefore beneficial for developing strategies to promote public health and health equity.

The home setting, especially parents, plays a vital role in the associations between SES and children’s screen time. According to socioecological models [13, 14], parents’ attitudes, beliefs, norms, and behaviors shape and create a shared social and physical environment in the home setting, and this environment affects children’s possibilities for different types of behaviors [13–15]. For example, higher parental self-efficacy to limit screen time is associated with less children’s screen time [16], whereas availability of media equipment and lack of physical activity (PA) equipment are associated with increased children’s screen time [17, 18]. Similarly, parental SES plays a role in shaping the home environment for children. Parental educational level is identified as an important indicator for SES that explains the differences in social and physical environment factors in the home setting [19, 20] because higher education is usually associated with greater understanding, capabilities, and skills to adopt healthy lifestyles compared to lower education levels [20]. For example, a recent study [21] reports that mothers with high education monitored their 6–8-year-old children’s television viewing time more frequently than mothers with low education. Mothers with high education also more often restrained from watching television in the presence of their children than mothers with low education [21]. Because preschool-aged children’s screen time occurs mostly at home [22], it is relevant to study further how the home setting mediates the associations between parental education and children’s screen time. This knowledge is valuable for the development of effective interventions aiming to diminish the SES gradient in children’s screen time.

Still, the mediation studies between parental education and children’s screen time are scarce. According to a recent review [19], the availability of media in bedrooms, portable play equipment, parental co-viewing, and parental modeling have most often been recognized as mediators in the associations between parental education and children’s screen time [19]. However, only one study was conducted among preschool-aged children [9]. Preschool age is a distinctive developmental period. Several habits and skills are learned during the preschool age, and the habits learned at this age tend to track into later childhood and adulthood [23–25]. During the early stages of development, preschool children are still highly dependent on the caregivers in their daily activity choices. As a result, parental influence could shape preschool children’s screen time more than school-aged children; thus, it is relevant to study the mediators of screen time in the home setting in the preschool-age group. The purpose of this study is to find out which factors in the home setting are mediators in the associations between parental education and preschool children’s screen time.

## Methods

### Study design

DAGIS (Increased Health and Wellbeing in Preschools) is a research project that aims to diminish SES differences in preschool children’s energy balance-related behaviors (EBRBs) (www.dagis.fi). As a part of this project, a cross-sectional study was conducted between September 2015 and April 2016 [26].

A total of 86 preschools (56% of those invited) in 8 municipalities in Finland agreed to participate in the study. The selection of municipalities for this study was based on SES indicators (larger variation of educational level, income level, and higher Gini coefficient) according to national statistics (Welfare Compass for monitoring regional welfare) by the National Institute of Health and Welfare [27]. Preschools were eligible to participate only if at least one of the preschool groups had 30% or more families agreeing to participate. All families with a preschool child aged 3–6 years were invited to participate in the study by an informational letter with a consent sheet attached. Preschools where less than 30% of families agreed to participate (*N* = 20) were excluded from the study, resulting in 66 participating preschools (39% of those invited). A total of 983 families (27% of those invited) gave written consent. Of them, 91 families were excluded because of a low consent rate in their preschool group and 28 families were excluded because of no data. In total, 864 children (26% of those invited) participated in the study. The study procedures were approved by the University of Helsinki Review Board in the humanities and social and behavioral sciences.

### Measures

#### Parental educational level

The educational level of the parent who completed the parental questionnaire was used as an indicator of parental SES in this study. The participating parents reported their highest education level from a ready-made seven-item list that covered the most common educational degrees in Finland. The answer options were categorized into three levels: low = high school or vocational school graduate or below, middle = Bachelor’s degree or equivalent, and high = Master’s degree or higher.

### Children’s screen time

Each parent completed a seven-day diary about their child’s sedentary behavior (*N* = 823). The translated-version of a previously validated diary was used [28]. This diary showed acceptable correlation coefficients with accelerometer measures and, therefore, was recommended for use as a measure of 3–5-year-old children’s sedentary behavior [28]. We modified the original version so that we asked separately television watching and DVD/video watching, and we added the use of tablet computers and smartphones as options. Parents reported if certain sedentary behaviors occurred on that day, how many times, and the total time of sedentary behavior on that day (in hours and minutes). Parents were asked to consider only the hours when the children were not at preschool. Only screen-time measures were used in this study. Screen time is a composite variable of the usage of television, computer, DVD/video, and tablet/smartphone. The reported total time of certain activities was transformed into minutes, and the daily minutes were added together. The weighted average of weekday (5/7) and weekend (2/7) screen time in minutes was calculated to form the daily average screen-time measure.

### Potential mediators

One parent in each family completed a questionnaire assessing the home environment related to the child’s EBRBs. The questionnaire was completed either online (57%) or in written form (43%). In addition, parents reported the children’s access to screens at home in the diary.

The development of this parental questionnaire was guided by the socioecological models [13, 14] and the adapted and modified socioecological framework of the DAGIS study [26]. The questionnaire measures aimed to capture the social and physical environmental factors in the home setting. The questionnaire was based on previous literature [29–31] and our own studies focused on the Finnish setting [32–35] and hypothesized to be associated with preschool children’s screen time. The following measures were used as potential mediators in this study: access to screens at home, descriptive norm for children’s screen time, satisfaction of children’s screen time, rules for limiting children’s screen time, parental screen time in front of children, parental importance for limiting children’s screen time, parental attitude toward societal pressures for screen time, and parental self-efficacy for limiting children’s screen time. The structure of these variables are presented in Table 1.

**Table 1:** Measures used to assess mediators in the home environment setting

Measures	Survey Items in the Parental Questionnaire	Scale and Range	Descriptives (N)
Access to screens at home^a^	Does your child have access to the following equipment at home?: television, DVD, video, tablet, smartphone, and computer.	Screen availability score was formed by summing all the equipment that the child had access to in the household.	Mean = 5 devices, SD = 1 device (*N* = 827)
Descriptive norm for children’s screen time	I think a suitable amount of daily screen time for children aged 3–6 years is a maximum of ____.	Open-ended answers in hours and minutes were transformed to minutes and treated as continuous in analyses.	Mean = 88 min, SD = 36 min
Satisfaction of children’s screen time	I am pleased with my child’s screen time.	The answer options ranged from strongly disagree (1) to strongly agree (5).	Mean = 4.0, SD = 1.0 (*N* = 795)
Rules for limiting children’s screen time	Do you have limits for how much time your child can spend a) watching television and b) using other electronic devices?	The three answer options were “yes,” “no,” and “don’t have the equipment.” This question was recoded so that “don’t have the equipment” answers [for the television *N* = 20 (2.5%) and for other equipment *N* = 15 (2%)] were set as missing values.	Yes (0) = 74% (*N* = 565), No (1) = 26% (*N* = 197)
Parental screen time in front of children	About how many hours per day do YOU use electronic devices during leisure time when your child is around? a) during weekdays and b) during weekends	Answer options (per day): 1 = none, 2 = less than 30 min, 3 = 30 min–1 h, 4 = 1–2 h, 5 = 3–4 h, 6 = 5 h or more. The items were recoded so that 1 = less than 30 min, 2 = 30–60 min, 3 = more than 60 min, and they were combined into one variable. This sum variable was recoded into three categories: 1 = less than 30 min, 2 = 30–60 min, 3 = more than 60 min.	Less than 30 min (1) = 33% 30–60 min (2) = 40% More than 60 min (3) = 27% (*N* = 796)
Parental importance for limiting children’s screen time (two items)^b^	It is important for me to limit my child’s screen time; I make sure that there are other activities available for my child to do instead of using electronic devices.	The answer options ranged from strongly disagree (1) to strongly agree (5). The statements were combined and divided by two.	Cronbach alpha = 0.30 Mean = 4.4; SD = 0.60
Parental attitude toward societal pressures for screen time (three items)^b^	There is pressure from society to purchase and use different electronic devices; sports as a hobby and the related costs (e.g., equipment, materials, subscription fees) are too expensive; it is important for my child to learn how to use electronic devices because I am not very good at using them myself.	The answer options ranged from strongly disagree (1) to strongly agree (5). The statements were combined and divided by three.	Cronbach alpha = 0.399 Mean = 2.8; SD = 0.80
Parental self-efficacy for limiting children’s screen time (three items)^b^	I find it difficult to limit my child’s screen time if he/she does not want it limited and is nagging; I find it difficult to restrain myself from using electronic devices when my child is around; I am concerned about my child’s use of electronic devices.	The answer options ranged from strongly disagree (1) to strongly agree (5). The statements were combined and divided by three.	Cronbach alpha = 0.462 Mean = 1.9, SD = 0.74

*SD* standard deviation

^a^Reported in the screen-time diary

^b^Based on loadings in the factor analysis

Access to screens at home, descriptive norm for children’s screen time, satisfaction of children’s screen time, rules for limiting children’s screen time, and parental screen time in front of children were assessed through questions that were adapted from previous studies [30, 31, 36]. Questions assessing parental importance for limiting children’s screen time, parental attitude toward societal pressures for screen time, and parental self-efficacy for limiting children’s screen time were sum variables formed by running factor analyses for items adapted from previous studies [29, 36] or added based on the results of the focus group interviews among Finnish parents [32]. Based on these factor analyses, the items loaded on the same factor were calculated together and divided by the number of items.

### Covariates

Children’s age and sex and season of measurement were used as covariates in the mediation analyses. Children’s age was continuous. Season of measurement covered three categories: early autumn (September–October), late autumn (November–December), and spring (January–April).

### Statistical analyses

The descriptive statistics and exploratory factor analysis was conducted by using the SPSS 23 statistical program (IBM SPSS Statistics: Chicago, IL). Internal consistency for multi-item scales was calculated using Cronbach’s alpha, and correlations were checked using Spearman correlations. The normal distribution of the screen-time variable was checked, and the outlier values of three standard deviations (SD) were removed (*N* = 4). The mediator models were conducted using Mplus 7.11 (Muthén & Muthén, Los Angeles, CA). Maximum likelihood estimation with robust standard error (MLR) was used as an estimator. In the mediator models, confidence intervals were adjusted for clustering at the family level (nested design of participating siblings from a family). The highest education group was treated as a reference category in the mediator models.

Mediation is defined as a causal model in which at least one independent variable is proposed as influencing an outcome through single- or multiple-intervening factors (mediators) [37, 38]. Our primary analyses were conducted in two stages. Firstly, in the mediator models, the individual associations of parental education on children’s screen time through each potential mediator (listed in Table 1) was checked (single-mediator models). The mediation hypotheses between parental education and preschool children’s screen time through home environment factors were tested by estimating the size of indirect association using a set of regressions and evaluating the statistical significance of the indirect association using confidence intervals. The effect of parental education on home environment factors (a-path in Table 3) and the effect of home environment factors on children’s screen time controlling for parental education (b-path in Table 3) were checked. The indirect effect (a*b), defined as the mediational effect in which parental education influenced the amount of children’s screen time through a certain home environment factor, was considered to be statistically significant if the confidence interval did not contain zero. Secondly, only the significant mediators from the single-mediator models were added simultaneously in the final model (multiple-mediator model). The total mediation effect and the independent mediation effects of the mediators in the multiple-mediator model were examined.

**Table 2:** The Spearman Correlation coefficients between the studied factors (listwise *N* = 610)

	1.	2.	3.	4.	5.	6.	7.	8.	9.
Outcome variable									
1. Children’s screen time									
Independent variable									
2. Parental educational level	−0.119**								
Potential mediators									
3. Access to screens at home	0.088*	0.024							
4. Descriptive norm for children’s screen time	0.344***	−0.099*	0.118*						
5. Satisfaction of children’s screen time	−0.299***	−0.048	−0.063	−0.157***					
6. Rules for limiting children’s screen time	0.100*	−0.011	0.026	0.198***	−0.153***				
7. Parental screen time in front of children	0.286***	−0.144***	0.008	0.290***	−0.165***	0.091*			
8. Parental importance for limiting children’s screen time	−0.246***	0.116*	−0.042	−0.222***	0.329***	−0.377***	−0.201***		
9. Parental attitude toward societal pressures for screen time	0.144***	−0.163***	−0.018	0.076*	−0.033	−0.075	0.050	0.029	
10. Parental self-efficacy for limiting children’s screen time	0.257***	0.065	0.050	0.096*	−0.453***	0.159***	0.281***	−0.272***	0.058

*p<0.05, **p<0.01, ***p<0.001

## Results

In total, 768 children (93% of those who returned diaries; 49% girls; mean age 4.7 years; standard deviation 0.89) had valid screen-time information for the analyses. Altogether, 11% of participants had at least one sibling who also participated in the DAGIS study. Of the participating children, 13% (*N* = 102) were only children and 61% (*N* = 485) had older sibling(s). Of the children who participated, 94% lived in two-parent families and 6% (*N* = 47) lived in one-parent families. Of the children, 44% (*N* = 354) participated in the DAGIS study in early autumn and 39% (*N* = 290) in late autumn. The rest of the children (20%, *N* = 164) participated in the spring of 2016. The children’s average daily screen time at home was 111 min (SD = 48.47), of which about 50% (56 min) was television viewing, 22% (26 min) was DVD/video use, 20% (22 min) was tablet/smartphone use, and 8% (9 min) was computer use.

A total of 808 parents responded to the questionnaire, and of these, 12% (*N* = 104) were fathers. The education level of the respondent parents (*N* = 792) was distributed in the following way: high education 29% (*N* = 233), middle education 42% (*N* = 327), and low education 29% (*N* = 232). The descriptive statistics of potential mediators are presented in Table 1.

The Spearman correlations between studied variables are presented in Table 2. Children’s screen time was significantly correlated with all of the other variables – the strongest correlation being with descriptive norm for children’s screen time (*r* = 0.344). Parental education level was significantly correlated with descriptive norm for children’s screen time (−0.099), parental use of screens in front of children (−0.144), parental importance for limiting children’s screen time (0.116), and parental attitude toward societal pressures for children’s screen time (−0.163).

### Single-mediator models

The total effect, direct effect, a- and b-path coefficients, and indirect effect with confidence intervals for the single-mediator models are presented in Table 3. The associations between parental educational level and potential mediators (a-paths) showed that a higher parental education level was associated with a lower descriptive norm, less use of screens in front of children, greater importance on limiting children’s screen time, and lower attitude toward societal pressures for screen time. The associations between potential mediators and preschool children’s screen time (b-paths) showed that all potential mediators have associations with preschool children’s screen time. Greater home access to screens, higher descriptive norm for children’s screen time, lower satisfaction of children’s screen time, not having rules for limiting children’s screen time, higher use of screens in front of children, less importance for limiting children’s screen time, higher parental attitude toward societal pressures for screen time, and lower parental self-efficacy for limiting children’s screen time were associated with children’s increased screen time.

**Table 3:** Mediation effect of home setting factors on association between parental education and preschoolers’ screen time^a b^

Potential Mediator (N)^b^	Parental Educational Level	Direct Effect β (95% CI)	a-path^c^ β (95% CI)	b-path^d^ β (95% CI)	Indirect Effect β (95% CI)^*^
Access to screens at home (*N* = 777)	Low	14.42 (5.14–23.71)	−0.06 (−0.29–0.16)	4.32 (0.88–7.76)	−0.27 (−1.28–0.74)
	Middle	7.10 (−1.08–15.27)	−0.00 (−0.21–0.20)		0.00 (−0.89–0.88)
	High (reference)				
Descriptive norms for children’s screen time (*N* = 788)	Low	9.36 (0.52–18.20)	9.72 (2.30–17.13)	0.48 (0.37–0.58)	**4.61 (1.03–8.19)**
	Middle	1.25 (−6.50–9.00)	11.84 (5.42–18.26)		**5.62 (2.25–8.99)**
	High (reference)				
Satisfaction of children’s screen time (*N* = 789)	Low	15.27 (6.16–24.37)	0.07 (−0.13–0.28)	−12.66 (−16.23 – −9.08)	−0.94 (−3.55–1.67)
	Middle	7.15 (−0.85–15.15)	0.01 (−0.16–0.19)		−0.18 (−2.39–2.03)
	High (reference)				
Rules for limiting children’s screen time (*N* = 784)	Low	14.22 (4.93–23.50)	0.01 (−0.08–0.10)	9.27 (0.89–17.66)	0.12 (−0.70–0.95)
	Middle	7.20 (−1.06–15.45)	−0.01 (−0.08–0.10)		−0.08 (−0.83–0.67)
	High (reference)				
Parental use of screens in front of children (*N* = 789)	Low	9.36 (0.50–18.22)	0.27 (0.12–0.42)	16.95 (12.44–21.45)	**4.61 (1.90–7.32)**
	Middle	3.75 (−4.24–10.45)	0.19 (0.05–0.33)		**3.28 (0.80–5.77)**
	High (reference)				
Parental importance for limiting children’s screen time (*N* = 786)	Low	10.95 (1.72–20.19)	−0.11 (−0.21 – −0.01)	−19.31 (−26.41 – −12.20)	**3.01 (0.65–5.48)**
	Middle	4.82 (−3.23–12.88)	−0.16 (−0.27 – −0.05)		2.17 (−0.04–4.31)
	High (reference)				
Parental attitude toward societal pressures for screen time (*N* = 788)	Low	12.44 (3.15–21.73)	0.34 (0.19–0.49)	5.85 (1.53–10.16)	**1.99 (0.34–3.63)**
	Middle	5.92 (−2.43–14.27)	0.15 (0.01–0.29)		0.89 (−0.15–1.93)
	High (reference)				
Parental self-efficacy for limiting children’s screen time (*N* = 789)	Low	15.95 (6.87–25.03)	−0.12 (−0.26–0.03)	16.73 (11.93–21.54)	−1.99 (−4.46–0.48)
	Middle	7.47 (−0.54–15.47)	−0.01 (−0.14–0.12)		−0.17 (−2.39–2.05)
	High (reference)				

^a^Single-mediator models

^b^Adjusted for children’s sex, children’s age, and season

^c^The associations between parental education and potential mediator

^d^The associations between potential mediator and children’s screen time adjusted with parental education

*Indirect effects in bold are statistically significant at p-level <0.05

The following factors had a significant indirect effect on the associations between parental education and children’s screen time (Table 3): descriptive norm for children’s screen time, parental use of screens in front of children, parental importance for limiting children’s screen time, and parental attitude toward societal pressures for screen time. Parents with high education had lower descriptive norm and used fewer screens in front of children compared to parents with middle or low education, and in turn, these factors were associated with lower screen time among children from parents with a higher education level. The indirect effect of descriptive norm was stronger among parents with middle educational level than low educational level, whereas the indirect effect of parental screen use was stronger among parents with low educational level than middle educational level. Parents with high educational level served as a reference category in both cases. Parents with high education placed greater importance on limiting children’s screen time and lower attitude toward societal pressures for screen time compared to parents with low education, and in turn, these factors were associated with lower screen time among children from parents with a higher education level.

### Multiple-mediator model

The results of the multiple-mediator model, including all significant single mediators simultaneously and the independent mediation role of each variable in the multiple-mediator model, are presented in Table 4. The associations of these mediators in the model remained similarly significant as in the single-mediator models.

**Table 4:** Mediation effect of home-setting factors on the association between parental education and preschoolers’ screen time^a^

Multiple-Mediator Model^b^	Parental Educational Level	Indirect Effect^*^ β (95% CI)
Total mediation effect (*N* = 791)		
All significant single mediators together	Low	**10.34 (5.86–14.82)**
	Middle	**8.66 (4.50–12.82)**
	High (reference)	
Independent mediation effect of the mediators in the multiple-mediator model		
Descriptive norm for children’s screen time	Low	**3.58 (0.73–6.43)**
	Middle	**4.37 (1.57–7.17)**
	High (reference)	
Parental use of screens in front of children	Low	**2.98 (1.06–4.90)**
	Middle	**2.11 (0.42–3.81)**
	High (reference)	
Parental importance for limiting children’s screen time	Low	**1.94 (0.17–3.71)**
	Middle	1.36 (−0.16–2.88)
	High (reference)	
Parental attitude toward societal pressures for screen time	Low	**1.85 (0.31–3.38)**
	Middle	0.83 (−0.14–1.79)
	High (reference)	

^a^Multiple-mediator model

^b^Adjusted for children’s sex, children’s age, and season

*Indirect effects in bold are statistically significant at p-level <0.05

## Discussion

This study aimed to identify home environment factors that mediate the associations between parental education and preschool children’s screen time at home. To summarize the main findings of this study, parents with high education had stricter descriptive norm for children’s screen time, used fewer screens in front of children, placed a greater importance on limiting children’s screen time, and felt less societal pressures for children’s screen time, and these home environment factors in turn were associated with lower screen time among their children.

Our findings highlight the importance of parental norms and attitudes toward preschool children’s screen time and how parental education shapes these norms and attitudes that influences children’s screen time. Current society increasingly promotes the importance of using screens, and screens have become a part of education and work life along with its entertainment role. When screens are available, screen-time reduction might be challenging and stressful for families with preschool children [39, 40]. Our study suggests that parents with low educational background feel more societal pressures regarding children’s screen time in the forms of the high costs of sporting activities, pressures to purchase and use different screens, and valuableness of learning to use screens at early ages. Motivation for changing their attitudes and norms because of possible health risks is not necessarily a priority among parents with low SES backgrounds [41, 42]. Parents with low educational backgrounds might place more value on children learning to use screens at an early age because they think it will enhance the children’s future possibilities in work life and school.

As higher education is usually associated with greater understanding, capabilities, and skills to adopt healthy lifestyles [20], parents with higher education backgrounds could have additional resources (e.g., finances, time) and capabilities to provide while limiting children’s screen time. Our results propose that parents with higher education backgrounds value the importance of limiting screen time more and can offer alternative options at home. However, we did not measure what these alternative options are and if they are sedentary in nature (e.g., quiet play, reading) or more activity-oriented behaviors (e.g., active play). The physical environment in the home setting could play a huge role in these alternative options. Previous studies have found that school-aged children with higher SES backgrounds have more PA equipment at home [43, 44], whereas school-aged children with lower SES backgrounds have more screen-related devices at home [43]. In addition, having a television in the child’s bedroom is an indicator of a higher risk for increased screen time and obesity [44, 45], and the presence of televisions in bedrooms is greater among children with low SES backgrounds [9, 44, 46]. We did not find any SES differences in the availability of screens in our study, but more research is needed to understand if the availability of PA equipment also plays a role in preschoolers’ screen time. These studies are relevant because several effective interventions aiming to diminish screen time in this age group have focused on increasing children’s activity levels [47].

Having rules regarding children’s screen time did not act as a mediator between parental educational level and children’s screen time. However, we did not ask what kinds of rules there are for limiting screen time. Other studies have found that parents who engage in screen-time less with their children are more likely to have stricter screen-time rules for their children [48–52]. Similarly, parents who perceive themselves as more efficacious in using screens report more often setting rules around their preschoolers’ screen time [53]. On the other hand, parents who are excessive screen users themselves are similarly strict with restrictions for their children but more often fail to follow the rules with their children and have joint screen time more often [48]. These results together with our findings underline the importance of parental self-control for limiting both their own and their children’s screen time. Future research could study more profoundly what kind of rules parents actually have for preschoolers’ screen time. It would be interesting to know, for example, if more permissive descriptive norms for screen time mean more permissive rules. A recent mediational study ascertained that parents with lower SES backgrounds had higher levels of permissive parenting practices toward sugared beverages, which was associated with higher consumption of sugared beverages [54]. Similarly, permissive descriptive norms for screen time from parents with lower educational backgrounds could mean more permissive parenting practices and rules, which in turn could mean preschoolers engage in more screen time.

Based on our study results, the future interventions aiming to diminish the socioeconomic gradient in preschoolers’ screen time should focus on developing strategies to tighten the descriptive norm for suitable screen time and to inform the suitable role of parental use of screens in front of children. A potential strategy could be that parents with lower educational backgrounds become aware of the difference between children’s actual screen time and their own descriptive norm about suitable screen time. This strategy is supported by another recent study, which concluded that greater parental consideration about an appropriate amount of screen time was associated with more actual screen time by preschoolers [5]. However, none of the previous interventions focused on preschool children’s screen time have used parental perceptions of the suitable amount of screen time as intervention strategies [55], but successful results have been achieved on other health promotion interventions focusing on changing norms [56]. Another potential strategy in future parental interventions and public health programs could be to raise the awareness of parents’ role modeling in screen use. A recent review recommended the encouragement of families in a “television turnoff week” as a good strategy to decrease children’s sedentary time [57]. The strategy could also work in preschoolers’ families if parents together with their children would limit the use of screens by replacing them with alternative options, such as doing something physically active together.

This study has some limitations that need to be discussed. The cross-sectional nature of the study limits the ability to identify the direction of the associations between home environment factors and children’s screen time. All the measures are based on the parent-reported answers, which are subject to either over- or under-reporting. In addition, differences might exist in how two parents report their children’s screen time. For example, a recent study suggests that in the case of a child’s energy intake, the information provided by fathers was more accurate compared to mothers [43]. Although we mainly adapted previously used questions, we do not have reliability or validity information on these measures in this sample. Some of the measures used in this study were developed during this project so validity information was not available for these measures. However, these items were mentioned to be important factors by Finnish parents of preschool children [32]. Our questions mainly focused on the social environment at home whereas some relevant physical environment factors, such as television in the children’s bedroom or PA equipment at the home, were not inquired. The sum variables in this study had low Cronbach alphas, but these sum variables included only a few statements [58]. In addition, the indirect effect sizes are generally small, but specific indirect effects in multiple-mediator models are usually attenuated to the extent that the mediators are correlated [59]. The low participation rate in this study limits the generalizability of these results. However, our sample represented a large sample of preschool children from diverse socioeconomic backgrounds. This variety allowed us to form equally distributed categories of parental education. Other indicators of SES, such as relative income, could bring additional knowledge of relevant mediators in the associations between parental SES and children’s screen time. Similarly, the mediators might also be device-specific. A recent meta-analysis [60] concludes that there still exists substantial heterogeneity for different domains of sedentary behavior and for the SES variable used between studies. Also, the associations between parental SES and screen time are contradictory between high- and middle-income countries so that in high-income countries, SES is inversely associated with screen time and television time whereas in low-middle-income countries, SES is positively associated with “other” screen time such as computers and videos [60].

A further strength of this study is that the information on children’s screen time was based on the seven-day diary, which is generally considered to be preferable to a questionnaire [61]. Rather than focusing only on television viewing, we measured children’s use of multiple screens. Thus, we were able to form a more detailed picture of overall daily screen time in this age group. The significance of a wide range of potential home environment mediators included in the analyses suggests that despite the relatedness to each other, these different home environment factors grab a somewhat distinct part of the explanation and should be considered in future intervention studies. More research is needed on the role of other possible environmental factors, aside from the home setting (e.g., the neighborhood), and their associations with parental SES and children’s screen time.

## Conclusions

This study found that factors related to parental norms and attitudes acted as mediators in the associations between parental education and children’s screen time. The lower screen time among children from parents with higher education was explained by the lower parental descriptive norm for screen time, greater parental importance on limiting screen time, lower societal pressures for children’s screen time, and less parental screen use in front of children. Interventions aiming to diminish socioeconomic gradient in preschoolers’ screen time should develop strategies that take into account these factors.

## Abbreviations

DAGIS: Increased Health and Wellbeing in Preschools
EBRB: Energy-balance-related behavior
MLR: Maximum likelihood estimation with robust standard error
SD: Standard deviation
SES: Socioeconomic status
